# Supplementary material for: The ExPAND Study: A Prospective Association Study into Endometriosis-Associated Pain, Neurosteroid Synthesis, and TRPM3
Source: Biomolecules. 2025 Sep 23;15(10):1352. doi: 10.3390/biom15101352 (PMC12563993; doi:10.3390/biom15101352)
Supplement: Supplementary file 1 [file biomolecules-15-01352-s001.zip › biomolecules-3855746-supplementary.pdf]

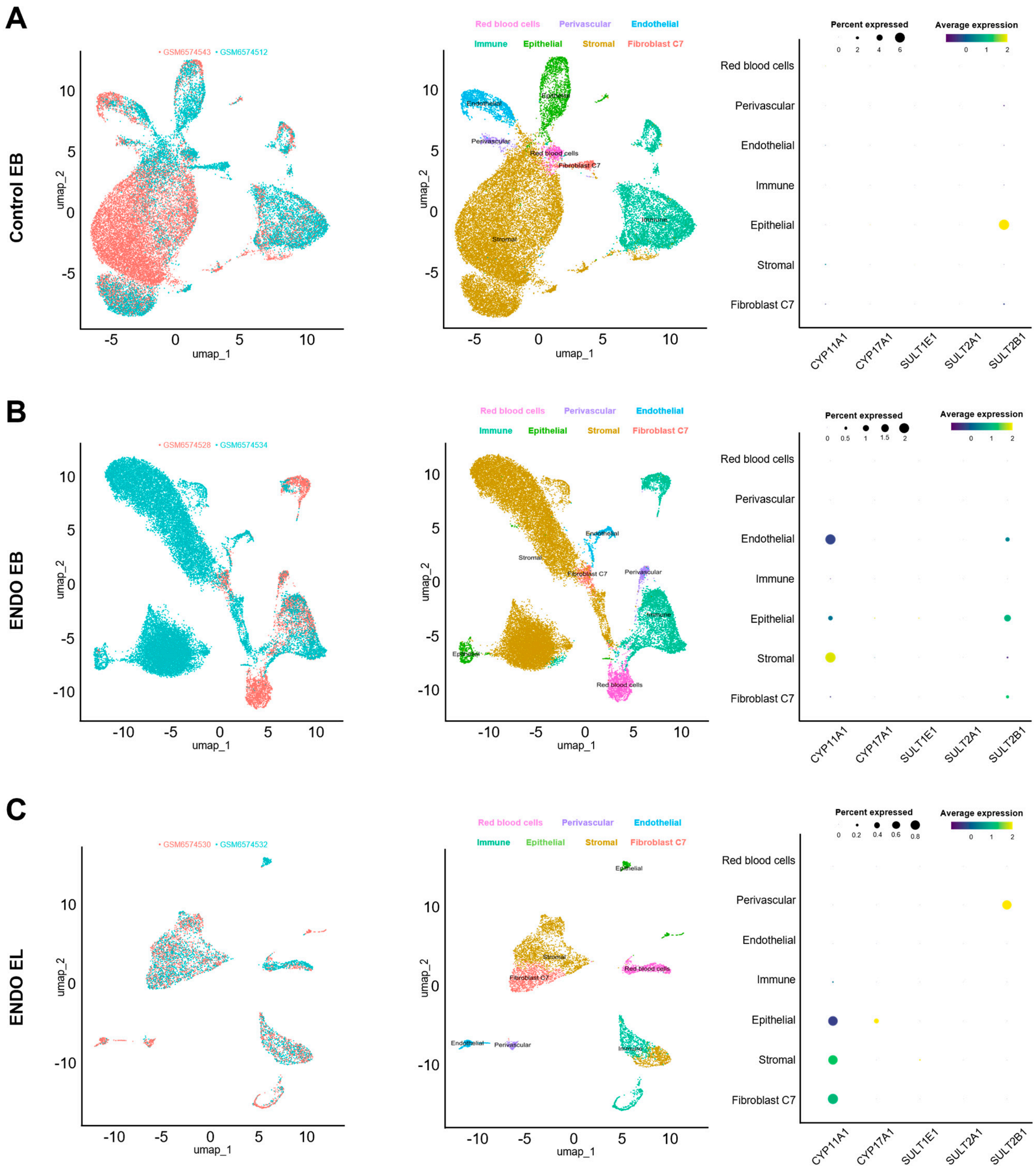

Supplementary Figure S1. Single cell RNA sequence analysis of available datasets corroborates RT-qPCR findings Analysis of the dataset by Fonseca et al. [11], including two healthy endometrium biopsies, two eutopic endometrium biopsies from patients and their paired ectopic endometriosis lesion (**A**,**B**,**C**, respectively). Dim plots of the merged datasets are provided as well as the annotated plots. Dot plots show the average expression and percentage of cells that express CYP11A1, CYP17A1, SULT1E1, SULT2A1 and SULT2B1 in different cell types.

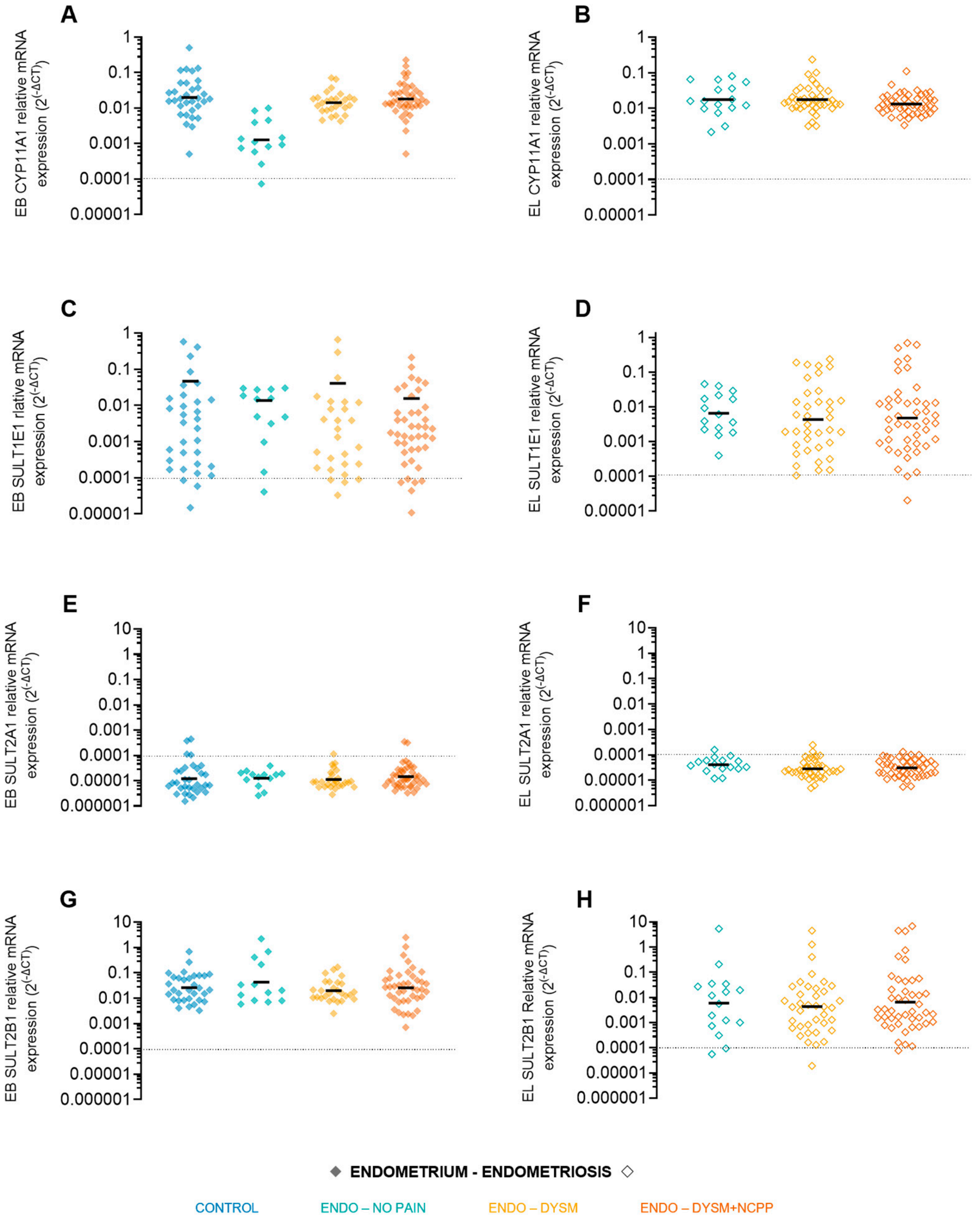

Supplementary Figure S2. mRNA expression of steroidogenic enzymes in the endometrium and deep endometriosis lesion of patients mRNA expression levels of members of the steroidogenic pathway in the endometrium and deep endometriosis lesion, respectively, of CYP11A1 (**A**-**B**), SULT1E1 (**C**-**D**), SULT2A1 (**E**-**F**), and SULT2B1 (**G**-**H**). These messenger RNA levels were quantified to the geometric mean of housekeeping genes GAPDH, HPRT1 and TBP. cDNA was synthesized from endometriosis lesions, sampled from the endometriosis patients without pain symptoms (Group 2, green), endometriosis patients with only DYSM (Group 3, yellow) and endometriosis patients with both DYSM and CPP (Group 4, red). Statistics: Outliers were detected using the ROUT method. Kruskal Wallis test with Dunn's correction was used for panel A, D, E, G and One-Way ANOVA with Tukey's correction for panel B, C, F, H. Data are presented as $2^{-\Delta CT}$ in a scatter plot together with an indication of geometric mean per group. Dotted line represents the threshold for physiological relevance.

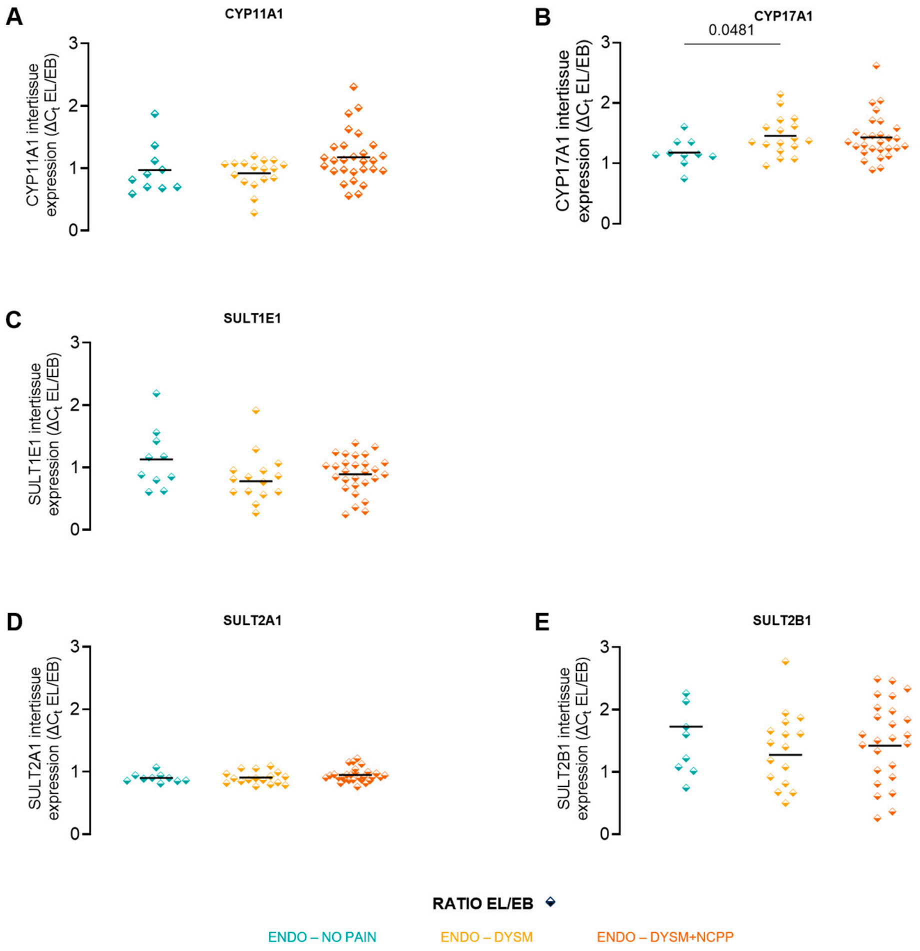

Supplementary Figure S3. Ratio of mRNA expression levels of members of the steroidogenic pathway in paired endometriosis lesion (EL) and endometrium biopsies (EB) EL/EB ratio of CYP11A1 (**A**), CYP17A1 (**B**), SULT1E1 (**C**), SULT2A1 (**D**), and SULT2B1 (**E**). Patients without pain symptoms (Group 2, green), endometriosis patients with only DYSM (Group 3, yellow) and endometriosis patients with both DYSM and CPP (Group 4, red). Statistics: Outliers were detected using the ROUT method. Kruskal Wallis test with Dunn's correction was used. For panel B, $\varepsilon^2 = 0.1044$, Group 2 *vs* Group 3, $p = 0.0481$, 95% $CI_{Group\ 2}$ [1.015-1.342], 95% CI_{Group3} [1.292-1.6.22]. Mean is indicated per group.

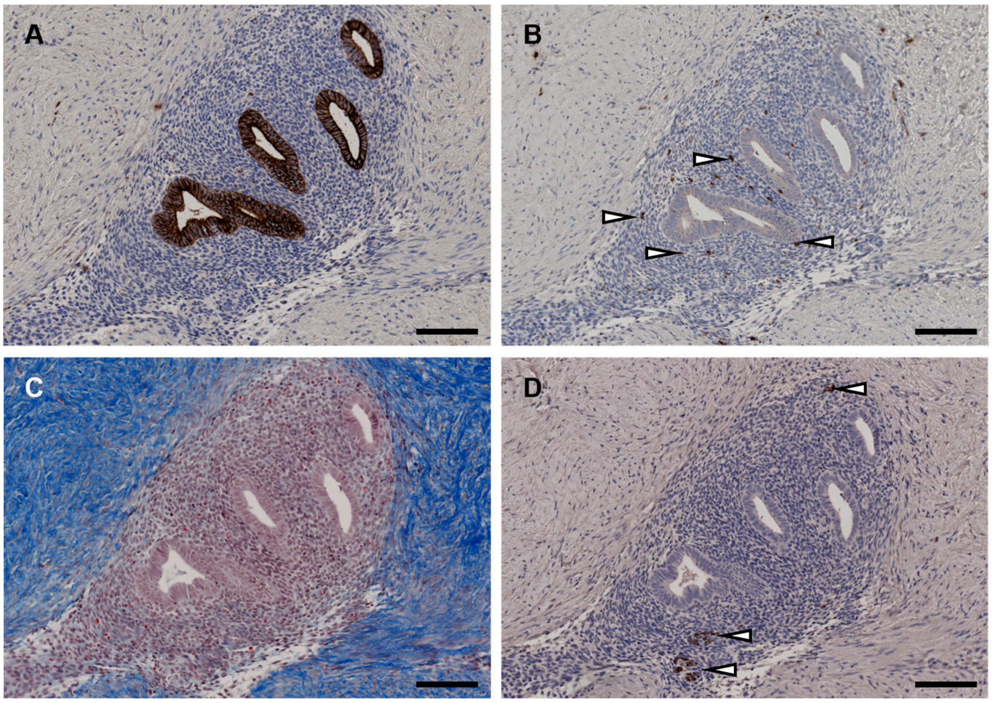

Supplementary Figure S4. Immunohistology stains of endometriosis lesions (**A**) Cytokeratin stain was used to identify the glandular structures of the endometriosis lesion. (**B**) CD68 stain was used to identify and quantify macrophages within the endometriosis lesion (indicated by white arrows). (**C**) Masson Trichrome stain was used to quantify collagen-rich areas (blue) *vs* cytoplasm (pink). (**D**) The presence of nerve fibres was investigated using a PGP9.5 stain (indicated by white arrows). Scale bar represents 100 µm.

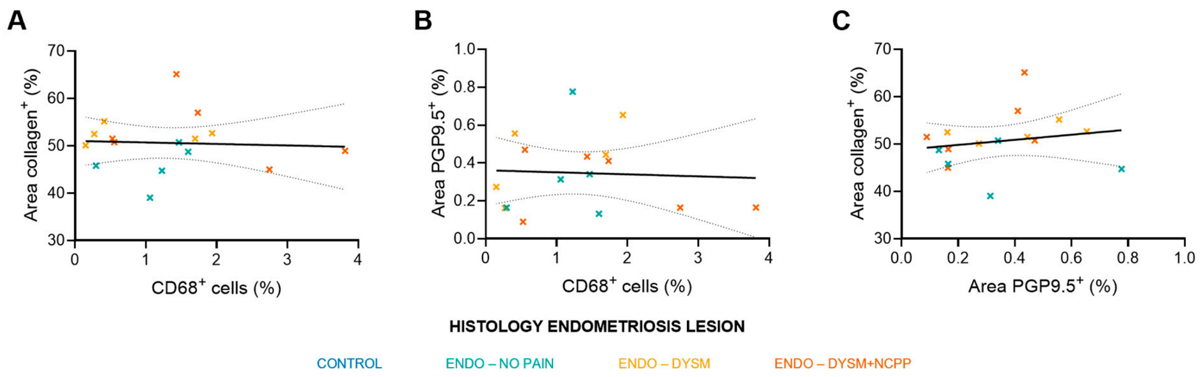

Supplementary Figure S5. Simple linear regression plots of inflammation markers and nerve density in endometriosis lesions (**A**) CD68+ cells *vs* collagen+ area, simple linear regression (Y = -0.31*X + 51.02; p=0.84). (**B**) CD68+ cells *vs* PGP9.5+ area, simple linear regression (Y = -0.01*X + 0.36; p=0.85). (**C**) PGP9.5+ area *vs* collagen+ area, simple linear regression (Y = 5.38*X + 48.74; p=0.49). Group 2 (n = 5; green), Group 3 (n = 5; orange) and Group 4 (n = 6; red).

	Group sizes			
	Group 1	Group 2	Group 3	Group 4
	n	n	n	n
**ENDOMETRIUM BIOPSY**				
qPCR - CYP11A1	32	13	27	42
qPCR - CYP17A1	32	13	27	42
qPCR - SULT1E1	32	13	27	42
qPCR - SULT2A1	32	13	27	42
qPCR - SULT2B1	32	13	27	42
**ENDOMETRIOSIS LESION**				
qPCR - CYP11A1		16	38	45
qPCR - CYP17A1		16	38	45
qPCR - SULT1E1		15	37	45
qPCR - SULT2A1		16	38	45
qPCR - SULT2B1		16	38	45
IHC		5	5	6
**PAIRED EL/EB**				
CYP11A1		10	17	28
CYP17A1		10	17	28
SULT1E1		10	17	28
SULT2A1		10	17	28
SULT2B1		10	17	25
**PERITONEAL FLUID**				
[PS]	35	19	36	60
[DHEAS]	41	21	36	68
**mRNA TO NEUROSTEROID EXPRESSION**				
CYP11A1 or SULT2B1 to PS		12	23	32
CYP17A1 or SULT1E1 to DHEAS		13	25	34

Supplementary Table S1. Sample sizes for analysis

**Gene name**	**Assay ID**	**RefSeq ID**	**Exon boundary**	**Assay location**	**Amplicon length**
**CYP11A1**	Hs00167984_m1	NM_000781.2	2-3	585	77
**CYP17A1**	Hs01124136_m1	NM_000102.3	6-7	1309	72
**SULT1E1**	Hs00193690_m1	NM_005420.2	2-3	260	109
**SULT2A1**	Hs00234219_m1	NM_003167.3	4-5	709	98
**SULT2B1**	Hs00190268_m1	NM_004605.2	1-2	351	80
**GAPDH**	Hs02758991_g1	NM_001256799.2	6–7	752	93
**HPRT1**	Hs02800695_m1	NM_000194.2	2–3	297	82
**TBP**	Hs00427620_m1	NM_001172085.1	2–3	578	91

Supplementary Table S2. List of used Taqman genes (Applied Biosystems)

		Component	Conc.	
Induction medium (1:1 N2 & B27 medium, spiked with small molecules	N2 medium	DMEM/F12 [1]		
		N2 supplement [1]	1X	
		Insulin [1]	5 µg·ml^{-1}	
		L-glutamine [1]	1 mM	
		MEM Non-essential Amino Acid Solution [1]	1X	
		β-mercaptoethanol [3]	90 µM	
		Penicillin [1]	50 U·ml^{-1}	
		Streptomycin [1]	50 mg·ml^{-1}	
	B27 medium	Neurobasal medium [1]		
		B27 supplement [1]	1X	
		L-glutamine [1]	1 mM	
		Penicillin [1]	50 U·ml^{-1}	
		Streptomycin [1]	50 mg·ml^{-1}	
	Small mol	LDN193189 [2]	1 µM	inhibitor of ALK1, ALK2, ALK3, and ALK6
		SB-431542 [4]	10 µM	inhibitor of ALK5, ALK4, and ALK7
		CHIR99021 [2]	5 µM	inhibitor of Glycogen Synthase Kinase 3
		DAPT [2]	5 µM	γ-secretase inhibitor
		SU5402 [4]	5 µM	VEGFR & FGFR inhibitor
Maturation medium		DMEM/F12 [1]		
		FBS [1]	10%	
		BDNF [2]	10 ng·ml^{-1}	
		GDNF [2]	10 ng·ml^{-1}	
		NT3 [2]	10 ng·ml^{-1}	
		NGF [5]	10 ng·ml^{-1}	
		Ascorbic acid [3]	200 µM	

Supplementary Table S3. hSCD neuron culture media components ([1]) Gibco, ([2]) STEMCELL Technologies, ([3]) Sigma-Aldrich, ([4]) Tocris Bioscience, ([5]) PeproTech

	CD68	Cytokeratin	PGP9.5
**Endogenous peroxidase block**	3% hydrogen peroxide in TBS buffer	3% hydrogen peroxide in methanol	3% hydrogen peroxide in TBS buffer
**Antigen retrieval**	pH 6 citrate solution at 95°C	0.04% pepsin in 0.01 M HCl at 37°C	pH 6 citrate solution at 95°C
**1st Antibody**	Anti-CD68	Anti-cytokeratin 8	Anti-PGP9.5
**Company-ref**	Abcam- ab955	Dako-Z0622	Dako-Z5116
**Marker**	Macrophages	Epithelial cells	Nerve fibres
**Type**	Mouse monoclonal	Rabbit polyclonal	Rabbit polyclonal
**Dilution 1st Ab**	1/500	1/500	1/1000
**Incubation 1st Ab**	ON; 4°C	2 h; 37°C	ON; 4°C
**2nd Antibody**	Goat anti-mouse PO labelled	Goat anti-rabbit PO labelled	Swine anti-rabbit HRP labelled
**Dilution 2nd Ab**	1/100	1/100	1/400
**Incubation 2nd Ab**	30′	30′	30′

Supplementary Table S4. Overview of immunohistological stain protocols

Supplementarty Code S1. Computational appendix (R scripts, key code snippets, and software version manifest)

All computational analyses were performed in R (version 4.3.1). The following packages were used: Seurat (version 5.0.1), harmony (version 1.2.0), ggplot2 (version 3.4.4), and viridis (version 0.6.5).

Data preprocessing

- Raw 10X Genomics outputs were read using Read10X().
- Separate Seurat objects were generated for each sample with CreateSeuratObject().
- Each object was normalized with NormalizeData() and variable features were identified with FindVariable-Features().

Integration and batch correction

- Objects were merged with merge().
- Batch effects across patients were adjusted with RunHarmony() using sample identity as the grouping variable.

Dimensionality reduction and clustering

- Principal component analysis (PCA) was performed with RunPCA().
- Cell-cell graphs were constructed with FindNeighbors() and clustering was performed with FindClusters(resolution = 0.5).
- UMAP embeddings were generated with RunUMAP() on Harmony-corrected dimensions.

Marker identification and cluster annotation

- Cluster-specific markers were identified using FindMarkers() with the following parameters:
    - min.pct = 0.25
    - logfc.threshold = 0.25
- Canonical lineage markers based on published papers (e.g., *EPCAM*, *KRT18*, *PECAM1*, *PDGFRB*, *CD14*) were visualized with DotPlot() and DimPlot().
- Clusters were relabeled into major cell lineages (Fibroblast C7, Stromal, Epithelial, Immune, Endothelial, Perivascular, RBCs) according to marker expression.

Visualization

- UMAP cluster maps were exported with ggsave() at high resolution (50 × 50 cm).
- Dot plots were styled with enlarged axis/legend text for publication quality figures.

Software manifest

- R: 4.3.1
- Seurat: 5.0.1
- harmony: 1.2.0
- ggplot2: 3.4.4
- viridis: 0.6.5
